# The dynamin inhibitor, dynasore, prevents zoledronate-induced viability loss in human gingival fibroblasts by partially blocking zoledronate uptake and inhibiting endosomal acidification

**DOI:** 10.1590/1678-7757-2024-0224

**Published:** 2024-09-30

**Authors:** Jacob KIRBY, Makayla STANDFEST, Jessica BINKLEY, Charles BARNES, Evan BROWN, Tyler CAIRNCROSS, Alex CARTWRIGHT, Danielle DADISMAN, Colten MOWAT, Daniel WILMOT, Theodore HOUSEMAN, Conner MURPHY, Caleb ENGELSMAN, Josh HALLER, Daniel JONES

**Affiliations:** 1 Indiana Wesleyan University Division of Natural Sciences Indiana United States Indiana Wesleyan University, Division of Natural Sciences, Indiana, United States

**Keywords:** Bisphosphonate-associated osteonecrosis of the jaw, Dynamins, Endosomes, Fibroblasts, Zoledronic acid

## Abstract

**Objective:**

For treatment of medication-related osteonecrosis of the jaw, one proposed approach is the use of a topical agent to block entry of these medications in oral soft tissues. We tested the ability of phosphonoformic acid (PFA), an inhibitor of bisphosphonate entry through certain sodium-dependent phosphate contransporters (SLC20A1, 20A2, 34A1-3) as well as Dynasore, a macropinocytosis inhibitor, for their abilities to prevent zoledronate-induced (ZOL) death in human gingival fibroblasts (HGFs).

**Methodology:**

MTT assay dose-response curves were performed to determine non-cytotoxic levels of both PFA and Dynasore. In the presence of 50 μM ZOL, optimized PFA and Dynasore doses were tested for their ability to restore HGF viability. To determine SLC expression in HGFs, total HGF RNA was subjected to quantitative real-time RT-PCR. Confocal fluorescence microscopy was employed to see if Dynasore inhibited macropinocytotic HGF entry of AF647-ZOL. Endosomal acidification in the presence of Dynasore was measured by live cell imaging utilizing LysoSensor Green DND-189. As a further test of Dynasore’s ability to interfere with ZOL-containing endosomal maturation, perinuclear localization of mature endosomes containing AF647-ZOL or TRITC-dextran as a control were assessed via confocal fluorescence microscopy with CellProfiler™ software analysis of the resulting photomicrographs.

**Results:**

0.5 mM PFA did not rescue HGFs from ZOL-induced viability loss at 72 hours while 10 and 30 μM geranylgeraniol did partially rescue. HGFs did not express the SLC transporters as compared to the expression in positive control tissues. 10 μM Dynasore completely prevented ZOL-induced viability loss. In the presence of Dynasore, AF647-ZOL and FITC-dextran co-localized in endosomes. Endosomal acidification was inhibited by Dynasore and perinuclear localization of both TRITC-dextran- and AF647-ZOL-containing endosomes was inhibited by 30 μM Dynasore.

**Conclusion:**

Dynasore prevents ZOL-induced viability loss in HGFs by partially interfering with macropinocytosis and by inhibiting the endosomal maturation pathway thought to be needed for ZOL delivery to the cytoplasm.

## Introduction

Medication-related osteonecrosis of the jaw (MRONJ) is a serious condition that affects both hard and soft jaw tissues. Zoledronate (ZOL), one of the causative agents of MRONJ, is often prescribed to patients with bone lesions associated with malignancies to prevent skeletal-related events and to reduce pain.^1,2^ These patients treated with ZOL, a nitrogen-containing bisphosphonate (NBP), had a significantly higher cumulative hazard of developing MRONJ than those treated with pamidronate or those treated sequentially with pamidronate and ZOL.^3^ In a study of multiple myeloma patients treated with bisphosphonates (BPs) in Greece, the risk of MRONJ increased with time of exposure to ZOL, as the cumulative hazard rose from 1% in the first year to 15% at four years of treatment.^4^ Multiple myeloma MRONJ patients in an Italian study had a significantly higher number of NBP infusions than those who did not develop MRONJ. The study identified the number of ZOL infusions as the most important risk factor.^5,6^ Woo, Hellstein and Kalmar^7^ (2006) revealed that patients receiving intravenous ZOL or pamidronate comprise 94% of published cases.

Human gingival fibroblasts (HGFs) are a widely accepted soft tissue cell model for the study of MRONJ. Multiple studies utilize HGFs for the characterization of MRONJ soft tissue effects.^8-10^ In one study, higher concentrations of ZOL resulted in impaired wound healing and death. These HGF effects were increased with the presence of bacterial lipopolysaccharide and mononuclear cell co-culture. In the mononuclear cell/HGF co-culture, increased levels of IL-1β, TNFα, and IL-8 were detected along with decreased IL-6 levels.^10^

Approaches to rescuing HGFs *in vitro* from ZOL-induced death include treatment with geranylgeraniol (GGOH), an intermediate of the mevalonate pathway that occurs downstream of the step catalyzed by farnesyl pyrophosphate synthase (FPPS). FPPS is inhibited by ZOL and other NBPs. Studies have indicated that low GGOH doses (0.5-10 μM) are minimally toxic to HGFs as well as oral keratinocytes.^11-14^ However, a study by Zafar, et al.^15^ (2014) revealed a loss in gingival fibroblast viability after a 50 μM dose of GGOH. Rattanawonsakul, et al.^13^ (2022) concerningly found that GGOH doses at 10 μM and above decreased metabolic activity in the immortalized oral keratinocyte line OKF6/TERT-2 as well as primary oral keratinocytes, potentially impacting mucosal healing. Their study suggested that some of the cytotoxicity was due to synergistic effects from the NBP and GGOH combination and that the inhibition of the mevalonate pathway was not the sole reason for cytotoxicity. These considerations indicate a narrow therapeutic window for GGOH, an undesirable characteristic for a clinical use compound. GGOH in myeloma patients could potentially stimulate cancer cell proliferation, contraindicating GGOH usage in patients with malignancy or at risk of developing malignancy.^13^ There is a clear need to find alternatives to GGOH as a topical treatment for MRONJ.

In a mouse model, swelling and cell necrosis consistently occurred after administration of NBPs, but phosphonocarboxylate rescue agents like phosphonoformate (PFA), phosphonoacetate (PAA), phosphonopropionate (PPA), and phosphonobutyrate (PBA) reduced the occurrence of both.^16^ Most of these rescue agents work to inhibit specific cellular proteins in the solute carrier family (SLC) and thereby prevent BP entry. Among the genes that code for these transporters are SLC20 (sodium-dependent transport of H_2_PO_4_^-^) and SLC34 (sodium-dependent transport of HPO_4_^2-^).^16-8^ These transporters are inactive in human osteogenic sarcoma Saos2 and murine calvarial MC3T3-E1 osteoblasts, but active in murine NIH3T3-3 fibroblast-like cells as well as C3H10T1/2 murine fibroblasts.^17^ Their expression in HGFs has not been determined. Interest in PFA to reverse NBP effects in oral soft tissue cells is warranted as well because PFA is already used clinically as FDA-approved Foscarnet/Foscavir. As a pyrophosphate analog, PFA inhibits viral DNA polymerases. This explains its use for the treatment of cytomegalovirus (CMV) and CMV-associated ophthalmic retinitis in AIDS patients who are resistant to gangcyclovir.^19^ PFA effects in NBP-treated HGFs have not been tested.

Pinocytosis (fluid-phase endocytosis) is another known mechanism of BP uptake into soft tissue that could potentially be blocked to thereby increase viability. NBP uptake is dependent on active endocytosis in some cells.^20^ Further, fluid-phase endocytosis is documented in HGFs.^21^ Internalization of NBPs in renal tubular epithelium through macropinocytosis was demonstrated by analysis with FITC-labeled dextran, an established marker for this effect. In addition, a greater uptake of ZOL is seen in cell types that have a high capacity for pinocytosis.^22^ Other studies have considered specific inhibition of macropinocytosis as a treatment for various pathologies.^23^ As membrane ruffling is a known first-step in pinocytosis, the dynamin inhibitor Dynasore is thought to inhibit this step through a dynamin-independent mechanism by remodeling actin filaments and decreasing plasma membrane cholesterol.^24^ To our knowledge, no articles are published to understand either ZOL uptake via HGF macropinocytosis or Dynasore effects on HGFs.

As ZOL is the most potent BP and a notable contributor to MRONJ, we chose to investigate strategies to limit ZOL death effects on a critical component of the oral cavity’s soft tissue, the HGF. Novel findings of this study include the fact that the SLC family of sodium-dependent phosphate transporters are not expressed in HGFs and consequently PFA does not rescue HGFs from ZOL death effects. In contrast, the dynamin inhibitor Dynasore does completely prevent ZOL-induced viability loss in HGFs. Dynasore appears to do this by partially blocking ZOL macropinocytosis and by inhibiting the acidification of HGF endosomes.

## Methodology

### Cell culture

HGFs were grown in fibroblast growth media containing 10% fetal bovine serum, proprietary fibroblast growth supplements, and 1% penicillin/streptomycin at 37 °C in a humidified, 5% CO_2_ incubator (cells and media components from ScienCell™, Carlsbad, CA, USA). HGFs were quickly thawed from frozen stock and seeded into a poly-L-lysine-coated T-75 flask (2 μg/cm^2^ poly-L-lysine, ScienCell™) with pre-warmed media. Media was replaced after 16 hours for unattached cell and residual DMSO removal and then every other day until cells reached 95% confluence. On reaching 95% confluence, cells were rinsed with calcium- and magnesium-free DPBS followed by trypsinization with 0.025% trypsin/EDTA. Trypsin was neutralized with ScienCell™ trypsin/EDTA neutralization solution and FBS, cells were centrifuged at 1000 rpm for 5 minutes, and the resulting pellet was resuspended in warmed media. An aliquot of the cell suspension was stained with 0.4% Trypan Blue in PBS to stain non-viable cells. Viable cells were counted and then seeded for experiments as indicated below. Cells were used for experiments within the first 3 passages.

### MTT viability assays

HGFs were seeded at 4,000 cells/well into uncoated 96-well plates and allowed to propagate for 24 hours before treatment. Each experimental condition was conducted in triplicate in two independent repeats. Concentrations of ZOL (Sigma-Aldrich, St. Louis, MO, USA; 10, 25, 50, 75, and 100 μM), GGOH (Sigma-Aldrich; 1, 10, 30, 50, and 100 μM), and PFA (Sigma-Aldrich; 0.025, 0.1, 0.5, 1, and 2 mM) were used. Vehicles for compound stocks were 0.1 N NaOH, 10% EtOH, and molecular biology grade water, respectively for each of ZOL, GGOH, and PFA. Cells were either exposed to ZOL by itself or the combination of ZOL and the compound for 72 hours. For Dynasore treatments, HGFs were exposed to Dynasore (Sigma-Aldrich; 10, 30, 50, 70, or 90 μM) or the corresponding DMSO vehicle for 15 minutes; then, the Dynasore or vehicle was removed, and either 50 μM ZOL or vehicle was added for 72 hours. At 72 hours of incubation at 37 °C in a humidified, 5% CO_2_ incubator, a total of 10 μl of MTT assay substrate, 3-(4,5-dimethylthiazol-2-yl)-2,5-diphenyl-2H-tetrazolium bromide purchased from MilliporeSigma (Burlington, MA, USA), was added to each well while the HGFs were incubated for 2.5 additional hours. Then 150 μl of acidified isopropanol was added to each well with thorough pipetting to dissolve the formazan product. Absorbance was measured using a BioTek EL808 microplate reader (630 nm absorbance subtracted from 570 nm absorbance; Winooski, VT, USA).

### Quantitative (real-time) RT-PCR (qRTPCR) analysis of SLC family transporter gene expression

RNA was isolated from HGFs using a Qiagen RNeasy Mini Kit^®^ according to manufacturer’s instructions following a QiaShredder homogenization (Qiagen, Germantown, MD, USA). RNA isolation effectiveness and purity was determined through both 1.2% non-denaturing agarose gel electrophoresis plus Agilent Technologies Cary 60 UV-visible (Santa Clara, CA, USA) and NanoDrop 2000 (Wilmington, DE, USA) spectrophotometry analyses. The agarose gels displayed clear bands for the housekeeping genes 18S rRNA and 28S rRNA with no degradation, indicating a high degree of purity. The RNA purity was also determined from spectrophotometry values for A260/A280 (2.00) and A260/A230 (1.96) ratios. These values demonstrated a high purity yield. Positive control RNAs (from human kidney and lung tissue) were purchased from Ambion (Austin, TX, USA). The purified, frozen RNAs were sent to Syd Labs, Inc. (Southborough, MA, USA) for qRTPCR analysis. The following sets of primers were designed by Syd Labs, Inc. and then synthesized by Integrated DNA Technologies (Coralville, IA, USA): Internal control human 18S rRNA gene (RN18S1), forward 5’GTAACCCGTTGAACCCCATT3’ and reverse 5’CCATCCAATCGGTAGTAGCG3’; human SLC20A1 gene, forward 5’CATCCTCCATAAGGCAGATCCAG3’ and reverse 5’TTGTCAAAGCCCAGCAACGG3’; human SLC20A2 gene, forward 5’AGACTCTCATGGCTGGGGAA3’ and reverse 5’ATGCAGTGCGTTCCTGAGAT3’; human SLC34A1 gene, forward 5’TAAGCGTTGCTGAGACCCAC3’ and reverse 5’CCAGCCTCTCTCCGTAGGAC3’; human SLC34A2 gene, forward 5’GTGTGCTAGTCCAGGCAGTT3’ and reverse 5’GGAAGACAAGAGCAGCAGGT3’; human SLC34A3 gene, forward 5’CAAAGTGGCCGGAGACATCTT3’ and reverse 5’AGCACGCCAATGACCAGTC3’.

Syd Labs, Inc. performed the remaining steps, starting with first strand cDNA synthesis (Syd Labs cDNA synthesis kit). First-strand cDNA synthesis involved a genomic DNA elimination reaction followed by a reverse transcription reaction for 15 minutes at 42 °C and a 3-minute incubation at 95 ºC to stop the reaction. The qRTPCR was executed with SYBR Green qPCR master mix/low ROX (carboxyrhodamine), first-strand cDNA template, and primers using the Agilent MX3000P real-time cycler and cycling program. Two independent experiments were each done in triplicate. A melting curve analysis of each resulting PCR product was done by heating the samples at 95 °C for 1 minute, 55 °C for 30 seconds, and then 95 °C for a minute in one cycle. Each qPCR product demonstrated a single peak. The ΔΔCt method was used for quantification of relative gene expression with the RN18S1 gene serving as the reference gene for normalization and analysis. Then each HGF gene expression value was set to one and the respective positive control gene expression value (from the RNA of either human kidney or lung tissue) was expressed as a fold-increase from the HGF gene expression value. Supplementary data figure S1 contains the experimental validation data.

### Visualization of ZOL-containing endosomes and FITC-dextran colocalization

HGFs were seeded at 23,750 cells/well in a 24-well plate on poly-L-lysine coated round glass coverslips (ThorLabs, Newton, NJ, USA) and grown overnight. The cells were preincubated for 15 minutes with either Dynasore vehicle or with 10 μM Dynasore. Coverslips were then incubated for 1 hour with 5 μM Alexa Fluor 647 (AF647)-ZOL (BioVinc, LLC, Pasadena, CA, USA) and 0.15 mg/ml FITC-dextran (Invitrogen Thermo Fisher, Waltham, MA, USA). Coverslips were fixed with 4.0% paraformaldehyde (Biotium, San Francisco, CA, USA) and then counterstained with 300 nM DAPI (Invitrogen Thermo Fisher). Then, they were mounted using ProLong™ Gold Antifade mountant (Invitrogen Thermo Fisher) and allowed to cure overnight before imaging. Images were captured on a Zeiss laser scanning 700 confocal microscope (Zeiss, Pleasanton, CA, USA) using the 63× oil immersion objective with the same microscope exposure parameters to allow comparison. The experiment was repeated twice.

### Endosomal acidification inhibition assay

HGFs were seeded at a density of 52,500/well into poly L-lysine-coated Lab-Tek™ chamber slides (Thermo Fisher, Waltham, MA, USA) 24 hours prior to the experiment. Cells were then treated with either 0.06 % DMSO (Dynasore vehicle) or 30 μM Dynasore, both in growth media, for 15 minutes at 37°C. Then LysoSensor Green DND-189 (Invitrogen Thermo Fisher) was added to each medium to bring the working concentration to 1 μM and incubated at 37 °C for 50 minutes. The last 5 minutes of that incubation included exposure to 40 μg/ml Hoechst 33342 dye (Invitrogen Thermo Fisher). Solutions were removed followed by a DPBS rinse. Then fresh growth media with either Dynasore vehicle or 30 μM Dynasore was added to the respective wells, both in the presence of ProLong™ Live Antifade Reagent (Invitrogen Thermo Fisher) for 1 hour at 37 °C. Live cells were then captured on a Zeiss LSM 700 confocal microscope at the same exposure using the 20× objective. Images were obtained of 100 cells per condition in each of two independent experiments. Then, image analysis was conducted in an unbiased, blinded approach with Image J software (NIH, Bethesda, MD, USA). For each cell the freehand tool was used to outline the cell fluorescence, then the Measure function from the Analyze menu was used to obtain the area and the mean fluorescence of the cell. The rectangle drawing tool was used to outline three different non-fluorescent regions around the given cell. Then, the area and mean fluorescence of each of these rectangles was obtained with the Measure function from the Analyze menu. The dynamin inhibitor, dynasore, prevents zoledronate-induced viability loss in human gingival fibroblasts by partially blocking zoledronate uptake and inhibiting endosomal acidification J Appl Oral Sci. 5/15 2024;32:e20240224 Corrected total cell fluorescence (CTCF) was calculated for each cell where area X mean fluorescence equals integrated density:  CTCF = integrated density of cell - average integrated density of cell's background .

### Determination of perinuclear endosomal localization: quantitation of intracellular fluorescence distribution

HGFs were seeded at 11,875 cells per poly-L-lysine coated round glass coverslip (ThorLabs), each contained in a well of a 24-well plate. Experiments were performed after 24 hours of growth. The positive controls were performed with TRITC-dextran (Invitrogen Thermo Fisher) as this marker undergoes fluid-phase endocytosis into endosomes that enter the endosomal-lysosomal maturation pathway. The experiments were repeated two independent times with each experiment containing the following treatments: a positive control of one hour exposure to 0.3 mg/ml TRITC-dextran subsequent to 15 minutes of pre-incubation with either DMSO vehicle or 30 μM Dynasore (235 total cells analyzed per condition) and a test of one hour exposure to 50 μM AF647ZOL subsequent to 15 minutes of pre-incubation with either DMSO vehicle or 30 μM Dynasore (216 total cells analyzed per condition). Following treatment, staining of nuclei was performed with 40 μg/ml Hoechst 33342 and staining of plasma membrane with 5 μM 3,3’-dioctadecyloxacarbocyanine perchlorate (DiO from AAT Bioquest, Pleasanton, CA, USA) prior to 4% paraformaldehyde fixation, mounting in ProLong™ Gold antifade reagent (Invitrogen Thermo Fisher), with a 12-hour curing process at room temperature in the dark. Images were captured with a Zeiss LSM 700 confocal microscope using the 20× objective. The 63× oil immersion objective was used to capture representative photomicrographs. Photomicrographs obtained with the 20× objective were analyzed to quantitate intracellular fluorescence distribution using an optimized CellProfiler™ software work pipeline (Broad Institute, Cambridge, MA, USA). Then, each photomicrograph image (.czi file format) was converted from multiple colors into greyscale images for each color by the Color to Gray module. Then the Identify Primary Object module identified the first object of interest, the nuclei. The next step in the pipeline was to use the Identify Secondary Object module to identify cells. Then, the Identify Tertiary Object module identified cytoplasmic regions of each cell in the image. The Measure Object Intensity Distribution module measured distribution of fluorescence intensity within each cytoplasm, reporting a mean fluorescence intensity for each of five different bins with the inner two bins (closest to the identified nucleus) designated as the perinuclear region. The Export to Spreadsheet module exported measurements of radial distribution intensities for each individual cell to an Excel spreadsheet. Mean Frac bin values were used to calculate the proportion of intracellular fluorescence that was perinuclear.

### Statistical analyses

Excel was used to calculate standard error of the mean. A two-tailed Student’s ttest using Microsoft Excel 2019 software (Microsoft Corporation, Redmond, CA, USA) was performed to evaluate significant differences between control and treatment groups. A p-value less than or equal to 0.05 was considered a significant difference.

## Results

### GGOH partially rescues and PFA does not rescue HGFs from ZOL-induced viability loss

**Figure 1:**
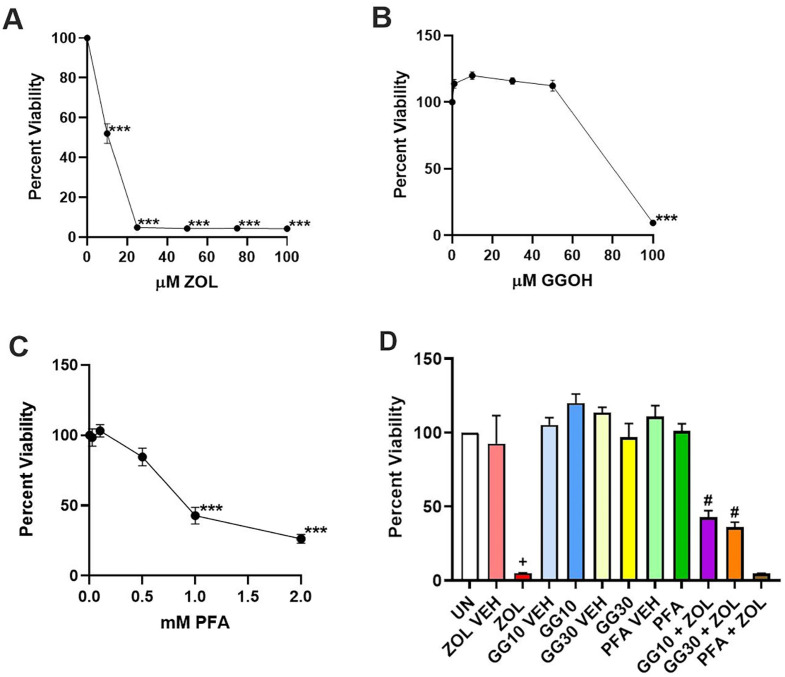
MTT viability assay for determining the doses of ZOL (A), GGOH (B), and PFA (C) to use in rescue assays. (D) ZOL-induced viability loss rescue assay results where UN indicates untreated (set to 100% viability), ZOL indicates 50 μM ZOL. GG10 and GG30 indicate 10 μM and 30 μM of GGOH, respectively. Error bars represent SEM with the double asterisk ** or the plus + indicating p less than or equal to 0.01 and the triple asterisk *** or the hash # indicating p less than or equal to 0.001. Significant difference comparisons include panels A-C: doses significantly lower than their respective vehicle control and panel D: ZOL significantly lower than ZOL vehicle plus each of GG10 + ZOL and GG30 + ZOL significantly higher than ZOL.

**Figure 3:**
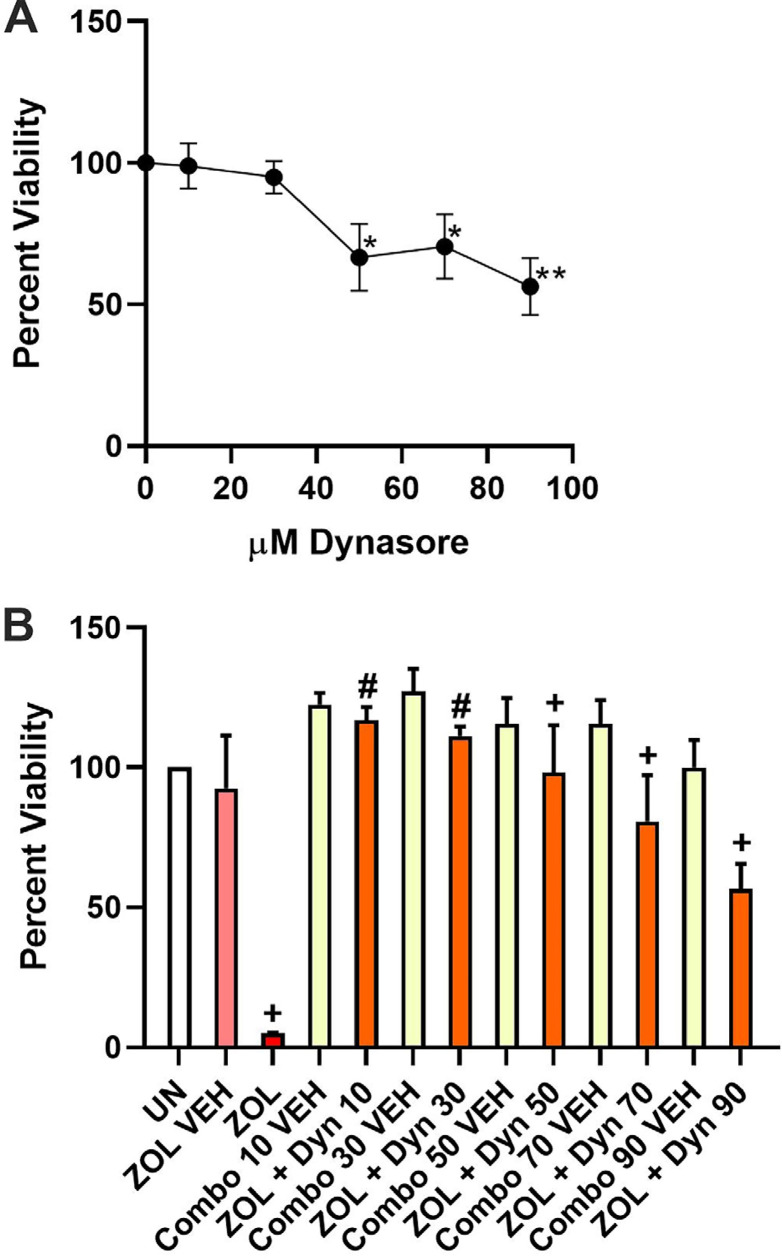
MTT viability assay to determine the toxicity of Dynasore in HGFs (A). (B) ZOL-induced viability loss rescue assay where UN indicates untreated (set to 100% viability) and the μM Dynasore concentrations are designated as Dyn 10, Dyn 30, etc. and ZOL is 50 μM in concentration. In panel B the corresponding combination of ZOL and Dynasore vehicles are designated as Combo 10 VEH, Combo 30 VEH, etc. Error bars represent SEM with triple asterisk *** or the plus + indicating p less than or equal to 0.001, double asterisk ** or the hash # indicating p less than or equal to 0.01, and single asterisk * indicating p less than or equal to 0.05. Significant difference comparisons include panel A: indicated doses significantly lower than their respective vehicle control. Panel B: ZOL significantly lower than ZOL VEH and each of the ZOL+ Dyn doses significantly higher than ZOL.

GGOH is known to rescue HGFs from ZOL-induced apoptosis.^14,15^ It is also known that in some cell types, PFA can prevent cellular entry of bisphosphonates and hence their subsequent death effects by inhibiting members of the SLC20/34 sodium-dependent phosphate transporter family.^16,17^ In a mouse ear-pinna model, PFA prevented necrotic and inflammatory reactions to nitrogen-containing bisphosphonates by inhibiting phosphate transporters.^16^ Although not effective against ZOL-induced viability loss in MC3T3-E1 and Saos2 osteoblastic cell lines, PFA did relieve ZOL-induced viability loss in NIH3T3 and C3H10T1/2 fibroblastic cell lines.^17^ We verified that GGOH behaved similarly in our research to other laboratory reports and also evaluated PFA for its ability to rescue ZOL-induced viability loss in HGFs. Dose-response curves of HGF viability following 72 hours of incubation in the presence of either ZOL, GGOH, or PFA were first determined (Figure 1 panels A, B, and C). The dose-response curves reveal that ZOL significantly reduces HGF viability to 52% at 10 μM (p<0.0005) compared to the vehicle control (100% viability). Of note, neither the ZOL vehicle or any of the other compound vehicles had a detrimental effect on HGF viability. The media-only control HGFs (UN for untreated) displayed a corrected absorbance of 0.33 ± 0.07, SEM while ZOL vehicle HGFs measured a corrected absorbance of 0.30 ± 0.06, SEM, p-value of 0.83 (data shown as percent viabilities relative to untreated set at 100% in Figure 1, panel D as well as Figure 3, panel B). At increasing doses of 25, 50, 75, and 100 μM ZOL, the viabilities decrease to 4.9%, 4.4%, 4.4%, and 4.3%, respectively (all p<0.0005 compared to vehicle control, Figure 1, panel A). A dose of 50 μM ZOL was determined as the treatment reference to allow rescue effect comparison with related studies. GGOH was tested at 1, 10, 30, 50, and 100 μM doses with both 10 and 30 μM doses significantly increasing viability to 120 and 116%, respectively, compared to vehicle control (both p<0.05, Figure 1 panel B). GGOH did not cause significant viability loss until 100 μM at which cells were only 9.5% viable (p<0.0005 compared to vehicle control, Figure 1 panel B). The highest concentration of GGOH that did not induce significant viability loss was 50 μM. Both the 10 and 30 μM GGOH doses chosen for testing of their rescue abilities are consistent with doses used in other studies. Based on literature values, 0.025, 0.1, 0.5, 1, and 2 mM doses of PFA were tested. Only at the 1 and 2 mM doses was significant viability loss detected at 42.9% and 26.4% (each p<0.0005 compared to vehicle control). HGFs were 84.6% viable in the presence of 0.5 mM PFA which did not reach significance compared to the 100% viable vehicle (p=0.06, Figure 1 panel C). Therefore, 0.5 mM PFA was the dosage chosen for rescue experiments.

An 18.3-fold decrease in viability occurred as measured by MTT assay on treatment of HGFs with 50 μM ZOL (p<0.01 compared to untreated, media control, Figure 1 panel D). In the same experiments, HGFs incubated with the combination of 10 μM GGOH and 50 μM ZOL or 30 μM GGOH and 50 μM ZOL displayed an 8.5-fold or 7.2-fold rescue, respectively, when compared to ZOL-treated cells (each p<0.0005, Figure 1 panel D). This partial rescue of ZOL-induced viability loss is reminiscent of values determined by others in HGFs.^14,15^ The optimized PFA concentration was tested in parallel for rescue with the combination of 0.5 mM PFA and 50 μM ZOL. PFA in combination with ZOL did not rescue as its viability was similar to that of ZOL by itself (Figure 1 panel D). In line with Baba, et al.^17^ (2019) in which their dose of PFA did not rescue murine osteoblast precursor MC3T3-E1 nor Saos2 osteoblastic cells from 10 μM ZOL effects, we found that non-cytotoxic PFA doses of 0.25-0.75 mM did not rescue primary human calvarial osteoblasts from 50 μM ZOL (data not shown). We also found that the same non-cytotoxic dose range of PFA was optimal for murine RAW264.7, RANKL-differentiated osteoclasts but did not rescue from 50 μM ZOL effects in these cells either (data not shown).

### Absence of viability loss rescue by PFA explained by lack of SLC transporter family expression in HGFs

**Figure 2:**
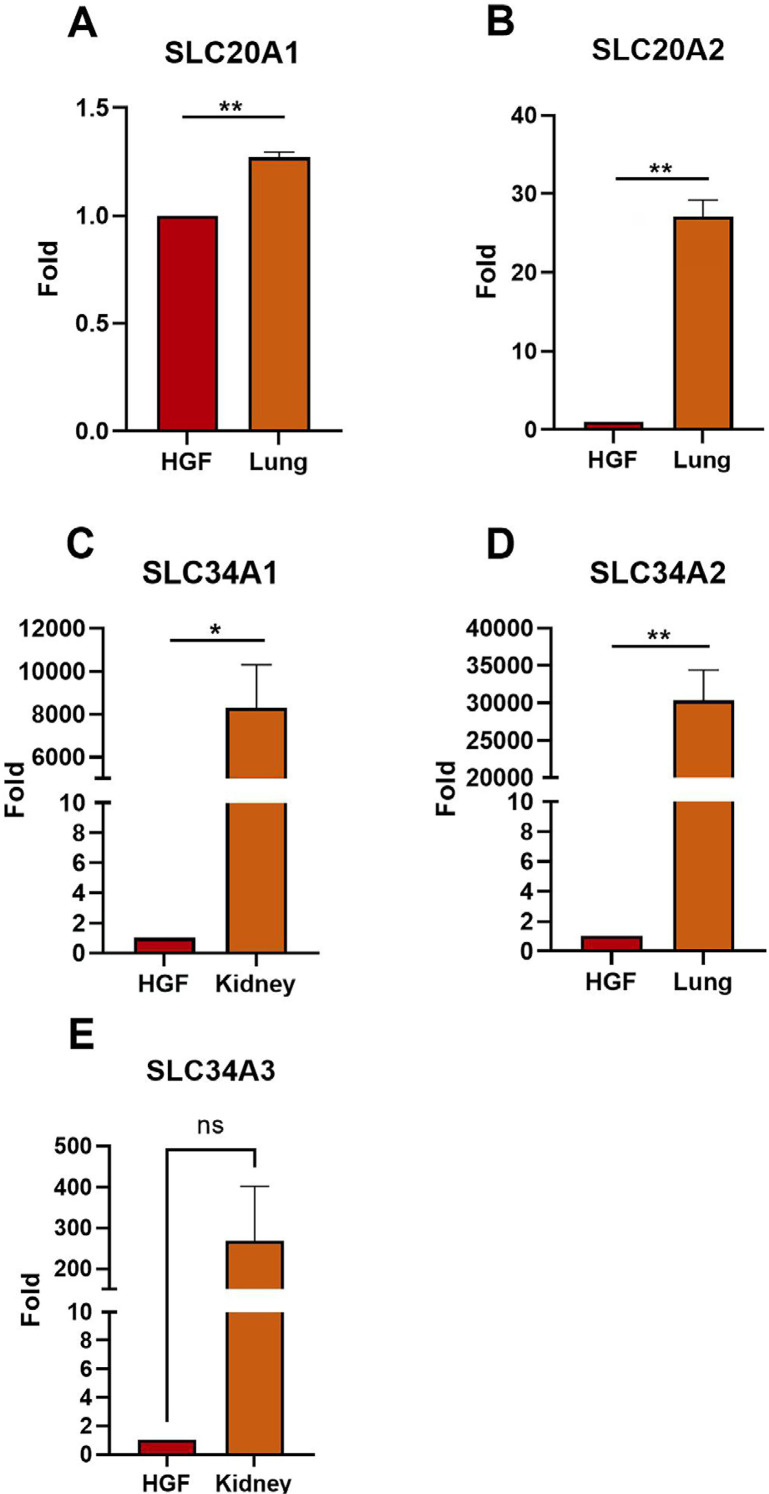
Quantitative real-time RTPCR to determine the relative expression of selected SLC genes in HGFs with 18S rRNA gene normalization. The relative amount of (A) SLC20A1, (B) SLC20A2, (C) SLC34A1, (D) SLC34A2, and (E) SLC34A3 transporter gene expression in HGFs compared to the respective, known positive control tissue expression is shown. In each graph, HGF expression is set to one with positive control expression as a fold increase. Error bars represent standard error of the mean. Significant differences between HGF and positive control are denoted as follows: double asterisk ** indicates p less than or equal to 0.01, single asterisk * indicates p less than or equal to 0.05, and ns indicates non-significant difference.

To address the absence of ZOL-induced viability loss rescue by PFA in HGFs, we investigated whether the members of the SLC20/34 sodium-dependent phosphate transporter family are expressed in HGFs. We analyzed this expression via quantitative real time RT-PCR. The SLC20A1 gene was moderately expressed, albeit significantly less than the lung tissue positive control (Figure 2 panel A). The SLC20A2, SLC34A1, and SLC34A2 genes were each minimally expressed compared to their positive controls with the latter demonstrating 27-fold, 8,290-fold, and 30,320-fold greater expression than the respective genes in HGFs (Figure 2 panels B, C, and D, respectively). Lung tissue was the positive control for SLC20A2 and SLC34A2 gene expression while kidney tissue was the positive control for SLC34A1 and SLC34A3 gene expression. Average SLC34A3 gene expression in kidney was 267-fold greater than the corresponding expression in HGFs (Figure 2 panel E). The combined expression data suggests that one or more alternative ZOL entry mechanisms other than the SLC20/34 transporters must exist in HGFs.

### The dynamin inhibitor, Dynasore, completely prevents ZOL-induced viability loss in HGFs

The inability of PFA to rescue and GGOH to only partially rescue prompted the investigation of another approach, the potential inhibition of macropinocytosis. Imipramine is a well-known macropinocytosis inhibitor.^23^ We exposed HGFs to 25 μM imipramine, the highest dose that did not result in any significant viability loss, for one hour prior to treatment with 50 μM ZOL. No rescue from ZOL-induced viability loss occurred (data not shown). Another compound known to inhibit macropinocytosis, Dynasore, was tested as a result. To determine an appropriate concentration of Dynasore for HGF exposure over a 72-hour treatment period, a dose-response curve was graphed based on the resulting viabilities from exposures to each of 10, 30, 50, 70, and 90 μM Dynasore. Both 10 and 30 μM Dynasore treatments did not significantly change viability compared to the vehicle control (Figure 3 panel A, p=0.90 and 0.42, respectively). A significant viability loss of 66.6% (p<0.05) occurred on the treatment with 50 μM Dynasore. Each of the 70 and 90 μM treatments led to significant viability losses (70.4 and 56.3%, respectively) when compared to the 100% viable vehicle control (Figure 3 panel A, p<0.05 and p<0.01, respectively).

Each of the Dynasore doses was tested for its ability to rescue or prevent ZOL-induced viability loss after 15 minutes of Dynasore pre-incubation followed by 72 hours of incubation in the presence of 50 μM ZOL. Pre-treatment with 10 μM Dynasore completely prevented viability loss, increasing viability by 23-fold compared to 50 μM ZOL treatment, as measured by MTT assay (Figure 3 panel B, p<0.001). In the presence of 10 μM Dynasore pre-treatment prior to 50 μM ZOL exposure, viability actually increased above the untreated condition (100% for untreated *versus* 116.9% in the presence of ZOL plus 10 μM Dynasore, p<0.05). The increasing doses of 30, 50, 70, and 90 μM Dynasore also increased viability significantly compared to 50 μM ZOL treatment by 22-, 19.4-, 16-, and 11.2-fold, respectively (Figure 3 panel B; p<0.001, p<0.01, p<0.01, and p<0.01, respectively). The 90 μM Dynasore dosage demonstrated prevention of viability loss, but this condition was significantly less viable than its respective vehicle control (Figure 3 panel B; “ZOL + Dyn 90” versus “ZOL VEH/Dyn 90 VEH,” with viabilities of 56.6% and 99.9%, respectively and p<0.01).

### Zoledronate and dextran colocalize inside the HGF in the absence and presence of Dynasore.

**Figure 4:**
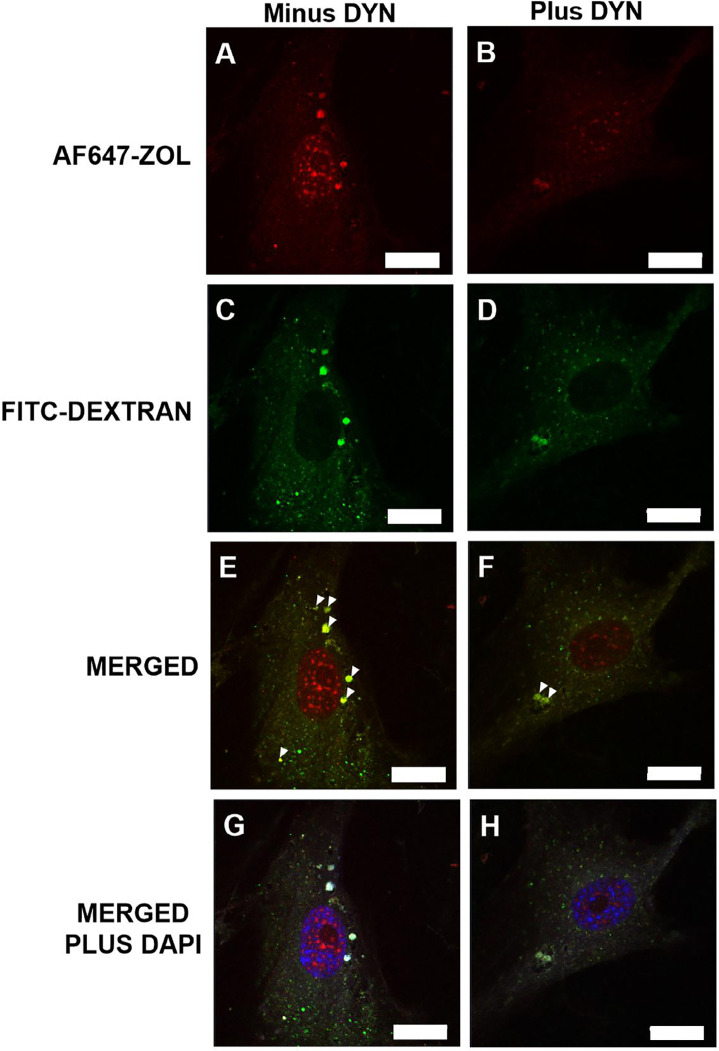
Confocal fluorescence microscopy to determine whether dextran and ZOL colocalize in HGFs. From the same experiment, a representative photomicrograph of an HGF in the absence of Dynasore (A, C, E, and G) and a representative photomicrograph of an HGF in the presence of Dynasore (B, D, F, and H) in which exposure settings were constant. The red-colored is AF647-ZOL, while the green is FITC-dextran, and the merged AF647-ZOL and FITC-dextran photomicrographs display the colocalizations highlighted by white arrowheads. The merged plus DAPI also includes the blue of the nucleus. 63X oil immersion objective, scale bar = 20 μm.

We hypothesized that Dynasore inhibited the membrane ruffling required for subsequent macropinocytotic uptake of ZOL into HGFs. To investigate this hypothesis on the mechanism by which Dynasore prevents ZOL-induced viability loss, we performed confocal fluorescence microscopy to visualize the entry of FITC-tagged dextran as well as Alexa Fluor 647-labeled zoledronate into HGFs grown on poly-L-lysine coated coverslips in both the absence and presence of Dynasore. HGFs were pre-incubated for 15 minutes with either Dynasore vehicle or with 10 μM Dynasore followed by a 1-hour incubation with 5 μM AF647-ZOL and 0.15 mg/ml FITC-dextran, a molecule known to enter cells via fluid-phase endocytosis. Photomicrographs were captured of cells from both conditions with constant exposure settings. In both the absence and presence of Dynasore, FITC-dextran colocalized with 5 μM AF647-ZOL. Colocalization to some of the endosomes is evident in the absence of Dynasore on comparing Figure 4 panels A and C to panel E. Colocalization in endosomes is marked by white arrowheads in panel E. Likewise, in the presence of Dynasore, endosomal colocalization is still evident on comparing Figure 4 panels B and D to panel F. Colocalization in endosomes is indicated by white arrowheads in panel F. The colocalization of these molecules suggests that ZOL enters HGFs through macropinocytosis; however, in this cell type, macropinocytosis is not completely inhibited by this concentration of Dynasore. The differing distribution of FITC- dextran and AF647-ZOL was noteworthy, as FITC-dextran did not get trafficked to the nucleus (Figure 4 panels C and D) but AF647-ZOL did (Figure 4 panels A, E, and G and to a lesser extent panels B, F, and H). Chaumet, et al.^25^ (2015) reported the delivery of *pseudomonas* exotoxin A from the cell surface of osteosarcoma MG63 cells to the nucleoplasm via nuclear envelope-associated endosomes. In addition, the intensity of both intracellular fluorescent ZOL and dextran were reduced in the presence of Dynasore while DAPI intensity remained similar.

### Dynasore inhibits endosomal acidification in HGFs

**Figure 5:**
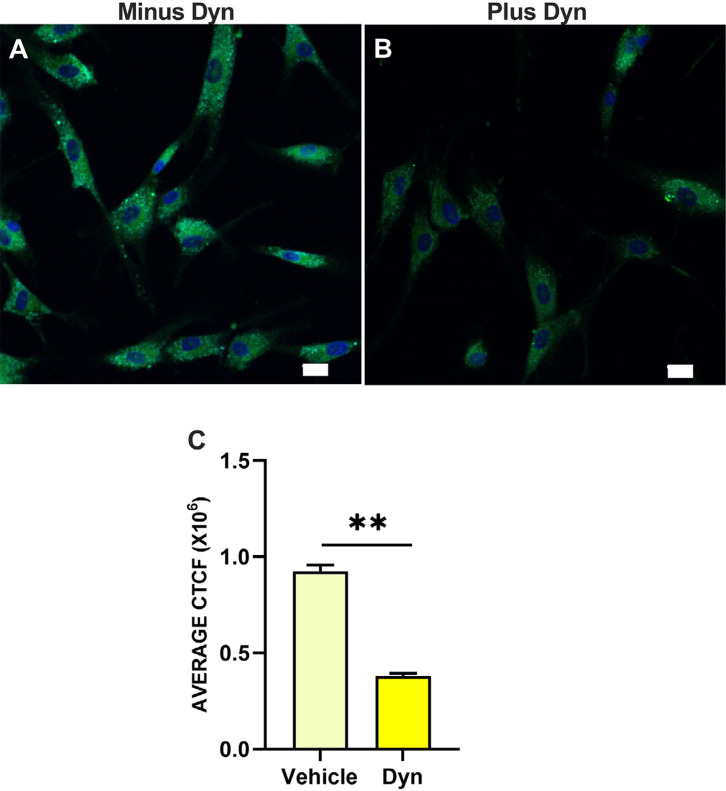
Confocal fluorescence microscopy of live HGFs exposed to LysoSensor Green DND-189 to measure endosomal acidification. Representative field of HGFs in the absence (A) and presence (B) of 30 μM Dynasore in which the exposure settings were kept constant. 20X objective, scale bar = 20 μm. (C) Image J quantitation of the average corrected total cell fluorescence (CTCF) where error bars represent SEM with double asterisk ** indicating p less than or equal to 0.01.

Mesaki, et al.^26^ (2011) showed that in HeLa cells, Dynasore blocks tubular endosome fission which in turn inhibits endosome acidification. Endosomal fission is thought to be an initial step in the endosomal maturation pathway, a pathway needed to deliver endosomal content for degradation and/or delivery to the cytosol. Thompson, et al.^27^ (2006) demonstrated the requirement of endosomal acidification following fluid-phase endocytosis for entry of bisphosphonates into the cytosol. Independently, Preta, Cronin and Sheldon^24^ (2015) reviewed the ability of Dynasore to inhibit endosomal acidification by blocking the endosomal V-ATPase in a dynamin-dependent manner. We therefore hypothesized that Dynasore exhibited ZOL-induced viability loss prevention in HGFs through inhibition of endosomal acidification. To test this hypothesis, HGFs were treated with either 30 μM Dynasore or with vehicle, followed by incubation in the presence of 1 μM LysoSensor^TM^ Green DND-189, a pH-sensitive probe specific for endosomes. With a pK_a_ of 5.2, this probe has limited fluorescence except when located inside acidic cellular compartments.^28^ Subsequent live capture of HGFs via confocal fluorescence microscopy using the same microscope settings for both Dynasore and vehicle-only conditions was followed by a blinded, unbiased measurement of corrected LysoSensor Green total cell fluorescence (CTCF). As seen in Figure 5 panel A, bright green fluorescence is observed in the live HGFs in the absence of Dynasore. That same fluorescence was reduced in the presence of Dynasore as observed in Figure 5 panel B. Results indicated that Dynasore decreased CTCF by 2.4-fold compared to vehicle control (p<0.005, Figure 5 panel C). From the live-cell imaging, it is apparent that Dynasore reduces endosomal acidification, a phenomenon which would interfere with delivery of ZOL to the cytosol or nucleoplasm, both cell compartments where FPPS is located.^29,30^

### Dynasore significantly decreases perinuclear localization of both TRITC-dextran-containing endosomes and AF647-ZOL-containing endosomes

**Figure 6:**
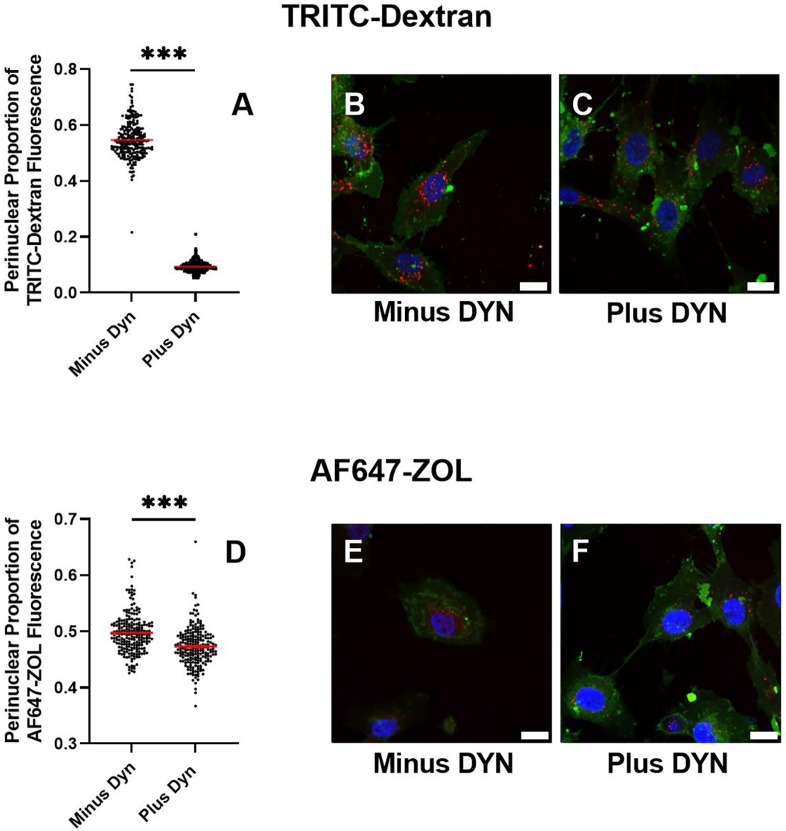
Measurement of the perinuclear localization of ZOL and the positive control for macropinocytosis, dextran, in the absence and presence of 30 μM Dynasore. Violin plots display the HGF perinuclear proportion of intracellular TRITC-dextran (A) and AF647-ZOL (D) fluorescence. Horizontal red lines indicate the average perinuclear proportion of intracellular fluorescence from all cells analyzed per condition via CellProfiler™ software. The triple asterisks *** indicate p equal to or less than 0.001. Representative 63× oil immersion objective photomicrographs of TRITC-dextran intracellular distribution in the absence (B) and presence (C) of Dynasore. Representative 63× oil immersion objective photomicrographs of AF647-ZOL intracellular distribution in the absence (E) and presence (F) of Dynasore. Scale bar = 20 μm.

Generally, as early endosomes mature to late endosomes and then to lysosomes, there is an expected pH drop from 6.5 to 5.5 and then to 4.5, respectively.^31^As the maturation process progresses, the positioning of late endosomes significantly becomes perinuclear.^26^ Delivery of ZOL to the cytosol and nucleus via the intracellular endosomal-lysosomal pathway has not previously been characterized. At least at the late endosome and lysosome pHs (5.5 and 4.5, respectively), ZOL is predicted to be fully protonated and released from the endosome; according to one study, within a 24-hour period.^32^We investigated the potential change in perinuclear localization of maturing, ZOL-containing endosomes as a result of Dynasore exposure by quantitating fluorescently-labeled ZOL distribution in the HGFs from confocal fluorescence photomicrographs utilizing a CellProfiler™ software pipeline. TRITC-labelled dextran was used as a positive control as dextran is known to undergo macropinocytotic uptake and trafficking through the endosomal-lysosomal pathway.^33^ After a 15-minute pre-incubation with either Dynasore vehicle or 30 μM Dynasore followed by a 1-hour incubation in the presence of TRITC- dextran, average TRITC-dextran perinuclear fluorescence was decreased from 54.4% (vehicle pre-incubation) to 9.3% (Dynasore pre-incubation) (p<0.001, Figure 6 panels A, B, and C). After a 15-minute pre-incubation with 30 μM Dynasore followed by a 1-hour incubation in the presence of AF647-ZOL, average AF647-ZOL perinuclear fluorescence was moderately decreased from 48.2% to 47.3% (p<0.001, Figure 6 panels D, E, and F). Dynasore’s reduction to perinuclear localization of AF647-ZOL is markedly smaller than its reduction to perinuclear localization of the TRITC-Dextran control. It remains to be determined whether ZOL interacts with Dynasore in the endosome or somewhere along the endosomal maturation pathway to interfere with or influence Dynasore function.

## Discussion

Inhibiting entry of ZOL into various cell types of the oral cavity is an attractive target for the prevention and treatment of MRONJ. Okada, et al.^34^ (2013) demonstrated that PFA decreased the inflammatory and necrotic effects of ZOL in mice. In contrast, PFA did not decrease these effects induced by the non-nitrogen-containing bisphosphonates oxidronate and medronate. Furthermore, PPi did decrease oxidronate and medronate effects. From these results the authors suggested that ZOL may enter soft tissue cells via ubiquitously expressed SLC 20 and/or SLC 34 transporters while oxidronate, medronate, and PPi may enter through SLC 17. PFA inhibits both the SLC 20 and SLC 34 sodium-dependent phosphate cotransporters but with a higher concentration (>10 mM) required for inhibition of the SLC 20 cotransporters.^35^ PFA is also known clinically as foscarnet, a pyrophosphate analog that interferes with the exchange of pyrophosphate from deoxynucleoside triphosphate as performed by herpesvirus DNA polymerase and HIV reverse transcriptase. This mechanism is behind the indication for PFA use in the treatment of these as well as other RNA and DNA viruses.^36^

We investigated PFA for its ability to rescue ZOL-induced viability loss in HGFs as an alternative to GGOH. As Baba, et al.^17^ (2019) reported that 20 and 100 mM PFA rescued both NIH3T3 mouse embryonic fibroblasts and mouse multipotent fibroblastic C3H10T1/2 cells from 10 μM ZOL, this was also a source of inspiration for this study. Our optimized 0.5 mM PFA dose for HGFs did not rescue these cells from 50 μM ZOL, which was explained by the lack of SLC 20A1-2 and SLC 34A1-3 transporter expression revealed by quantitative real time RT-PCR. We did not attempt to use PFA in ZOL-treated oral keratinocytes. Literature searches for each of “SLC 20” and “SLC 34” in conjunction with “oral keratinocyte” did not yield any results. The only related transporter we found on searches was the SLC 38A1 transporter that is expressed in oral epithelium. This glutamine importer is upregulated in oral squamous cell carcinoma tissue.^37^ As such, analysis of expression of the SLC 20 and 34 transporters in oral keratinocytes is necessary.

In macrophages, NBPs appear to be taken up by fluid-phase endocytosis.^27^ More specifically, ZOL enters renal tubular cells via fluid-phase endocytosis.^22^ As such, our investigation focused on the inhibition of macropinocytosis in HGFs and whether this would prevent ZOL entry. Lin, et al.^23^ (2018) systematically screened 640 FDA-approved drugs for their ability to specifically inhibit macropinocytosis. Their results identified imipramine (a tricyclic antidepressant) as the most promising macropinocytosis inhibitor. Imipramine appeared to act by inhibiting the first step of macropinocytosis, membrane ruffle formation. Lin, et al.^23^ (2018) pre-treated with 5 μM imipramine for 60 minutes and then stimulated the cells with 1 μM PMA for 2 hours in the presence of FITC-dextran to observe macropinocytosis inhibition in 4T1 breast cancer and immature dendritic cells as well as RAW264.7 macrophages. Our use of an optimized dose (25 μM) of imipramine for one hour prior to 50 μM ZOL incubation did not rescue HGFs. Unlike Lin, et al.^23^ we chose not to use phorbol esters to stimulate macropinocytosis as the physiological relevance of these tumor promoters is unclear in the context of our experiments.

Another prevention of ZOL soft tissue cell entry was revealed by Zlatev, et al.^20^ (2016) T47D and MCF-7 breast cancer cell lines were exposed to either plain ZOL, calcium-complexed ZOL, or liposome-encapsulated ZOL with and without inhibitors for different endocytosis mechanisms. Regardless of the ZOL formulation, its uptake in these breast cancer cells relied on dynamin. Dynasore, the inhibitor of dynamin GTPase activity needed for clathrin-coated endocytic vesicle scission from the plasma membrane, was used for a 15-minute pre-incubation at a concentration of 80 μM by these authors. For uptake measurements, they used 10 μM [^14^C]-ZOL. In our HGFs, this concentration of Dynasore caused significant viability loss, so 30 μM was the maximal concentration we employed for confocal fluorescent microscopy colocalization analysis. In our study, 30 μM Dynasore prevented 50 μM ZOL-induced viability loss. Although the different concentrations of ZOL and Dynasore used in various studies limited comparison, the uptake effects observed in breast cancer cells were different. In HGFs, Dynasore did not completely prevent entry of AF647-ZOL nor FITC-dextran. In MCF-7 breast cancer cells, Dynasore elicited a 20-fold decrease in intracellular ZOL accumulation compared to control.^20^ A limitation to our study is the use of 50 μM ZOL for the viability rescues, as this dose is significantly above the 0.4-5 μM level thought to be present in plasma and the oral mucosa after intravenous infusion.^9^ However, multiple other studies with HGFs also use 50 μM ZOL, thus allowing for more direct comparison.^14,15^A promising finding is the fact that only 10 μM Dynasore completely prevented HGF viability loss at this elevated level of ZOL.^14,15^

The reported multitude of Dynasore’s effects complicates its use as a way to prevent ZOL-induced cell death in oral tissues. Dynasore inhibits the GTPase activity of dynamin as well as the vesicular H^+^-ATPase that is needed to pump protons into endosomes to acidify them. This is a critical, dynamin-dependent step in the endosomal maturation and trafficking pathway.^24^ Two other dynamin-dependent effects include disruption of cholesterol distribution in the cell by inhibiting LDL-cholesterol movement from the endolysosomal network to the endoplasmic reticulum and inhibiting the amount of cholesterol that is trafficked from the endoplasmic reticulum to the Golgi apparatus. In turn, Dynasore has the dynamin-independent effect of reducing the cholesterol of the plasma membrane to disrupt lipid raft organization and to inhibit membrane ruffling required of macropinocytosis.^24^ Dynasore is known to bind serum proteins and detergents which reduces its inhibitory activity in both *in vivo* and *in vitro* applications. As a result, analogs of Dynasore have been synthesized to reduce detergent binding and cytotoxicity as well as increase potency. These dihydroxyl and trihydroxyl analogs are the Dyngo compounds.^24,38,39^

The multiple cell physiologies that Dynasore influences suggests that the compound is not ideal to pursue as a treatment for MRONJ. A compound with more directed, less peripheral effects is desirable; however, the proof of concept shown here suggests that endosome acidification inhibitors are worth screening in HGFs for their effectiveness and specificity. As such, Niclosamide, an FDA-approved anti-helminthic drug, emerges as an endosomal inhibitor of interest. In a screening of six FDA-approved drugs that act similarly to the endosomal acidification inhibitors bafilomycin A1 and NH_4_Cl, 10 μM Niclosamide had the greatest effect of impeding SARS-CoV2 virus entry into HEK-293T cells. Even at 2.5 μM dosage, Niclosamide displayed late endosome pH neutralization effects.^40^ Acidification of the early endosome is needed for the internalization of SARS-CoV2 via the clathrin-independent carriers (CLIC)-GPI anchored protein enriched early endosomal compartments (GEEC) pathway.^40^ Further studies in HGFs and other oral tissue cells are needed to investigate endosomal acidification inhibition drugs in the presence of ZOL. Whether the clathrin- and dynamin-independent but pH-dependent CLIC/GEEC endocytic pathway is a significant ZOL entry route requires further experimentation.

## Conclusions

Consistent with the results of others, we observed GGOH rescue of ZOL-induced viability loss in HGFs; however, the rescue was partial. We investigated other approaches to accomplish this rescue, because GGOH as a candidate topical agent for MRONJ is limited by a potential narrow therapeutic range and the possibility of triggering tumorigenesis.^13^ PFA was also tested in parallel but failed to rescue as the targets of the PFA inhibitor, the SLC sodium-dependent phosphate transporters, were not expressed in HGFs. This novel finding prompted the trial of the dynamin inhibitor Dynasore for its ability to prevent ZOL-induced viability loss in HGFs. In another novel result, a 15-minute pre-incubation with Dynasore completely prevented ZOL-induced viability loss in HGFs. ZOL colocalized with dextran in both the absence and presence of Dynasore, which indicate that Dynasore does not completely inhibit macropinocytosis to the degree of preventing ZOL entry; however, Dynasore did inhibit acidification of HGF endosomes and their maturation to the perinuclear region. This proof-of-concept should foster further investigation of compounds that are specific endosome acidification inhibitors in HGFs.
